# A modular risk management framework for integrating cybersecurity and patient safety in medical device software development

**DOI:** 10.3389/fdgth.2026.1903787

**Published:** 2026-09-29

**Authors:** Pekka Pohjosaho, Tommi Mikkonen, Kyriakos Stefanidis, Vlad Stirbu, Tuomas Granlund

**Affiliations:** 1 Solita Oy, Tampere, Finland; 2 University of Jyväskylä, Jyväskylä, Finland; 3 I.S.I.—Industrial Systems Institute/ATHENA R.C., Patras, Greece; 4 Tampere University, Tampere, Finland

**Keywords:** application lifecycle management, cybersecurity, medical device software, modular risk schema, patient safety, risk management, secure by design, traceability

## Abstract

Medical device software operates in increasingly connected environments where cybersecurity incidents can directly compromise patient safety. While established safety risk management standards such as ISO 14971 provide mature frameworks for managing unintended hazards, they implicitly assume non-malicious causes and that risks are amenable to probability estimation. Cybersecurity risk management, by contrast, addresses adversarial threats, exploitability, and inherent uncertainty, requiring fundamentally different vocabularies, risk models, and acceptance criteria. In practice, organisations often force these domains into a single monolithic risk structure, producing terminological confusion, incommensurable risk values, and siloed assessments with no mechanism to escalate findings between safety and security processes. This paper presents the Modular Risk Schema Framework, a concrete, repeatable approach to integrating safety and cybersecurity risk management in medical device software development. Rather than forcing divergent domains into a unified risk model, the framework supports multiple domain-specific risk schemas in parallel, each with its own information model, risk model, acceptance criteria, and level of rigor, connected through explicit traceability interfaces. These interfaces ensure systematic escalation of safety-relevant cybersecurity risks and identification of safety controls with security implications. The framework is embedded within a single quality management system, enforces security by design through phase-gated deliverables aligned with the software lifecycle, and is designed for implementation in modern application lifecycle management environments without dependence on specific tools. The approach has been developed and applied in industrial contexts and aligns with the requirements of ISO 14971, IEC 62304, IEC 81001-5-1, and current regulatory guidance.

## 1 Introduction

Modern healthcare increasingly relies on interconnected health information systems, cloud-based services, connected medical devices, and software as a medical device (SaMD) to enable real-time patient data exchange, remote monitoring, and clinical decision support. These developments offer substantial benefits in terms of access to care, operational efficiency, and quality of patient outcomes (1). However, this connectivity fundamentally alters the risk landscape for medical devices: cybersecurity incidents are no longer confined to data confidentiality breaches or technical service disruptions but can lead directly to safety-critical failures—delayed or missed alarms, corrupted clinical data, loss of essential device functions, or delivery of incorrect therapy (2). Medical device software, whether embedded in a physical device or operating independently as SaMD, represents a particularly critical intersection of these concerns, as it is simultaneously subject to cybersecurity threats inherent in networked software systems and to patient safety obligations inherent in its clinical purpose.

The established regime for managing safety risks in medical devices is centred on ISO 14971, which defines a systematic process for identifying hazards, estimating and evaluating associated risks, controlling those risks, and monitoring the effectiveness of controls throughout the product lifecycle (3). For software-based devices, IEC 62304 complements this by specifying software lifecycle activities whose rigor is scaled according to the software’s safety classification (4). The core analytical vocabulary of this regime (hazards, hazardous situations, harms, and the estimation of risk as a combination of probability of occurrence of harm and severity of that harm) is well understood, widely applied in industry, and closely aligned with regulatory expectations worldwide. The framework supports benefit–risk evaluation, allowing acceptance of residual risks where they are outweighed by expected clinical benefits. However, it implicitly assumes that the causes under analysis are non-malicious (design errors, component failures, foreseeable misuse, environmental conditions) and that the probability of harm can be meaningfully estimated from historical data, analytical models, or expert judgement.

Cybersecurity risk management for medical devices is addressed by a growing body of standards and regulatory guidance, including IEC 81001-5-1, the IMDRF Principles and Practices for Medical Device Cybersecurity (2), MDCG 2019-16 (5), and the FDA premarket cybersecurity guidance (6). These documents collectively emphasise that cybersecurity is integral to device safety, must be managed within the quality management system, and should follow a total product lifecycle approach. The domain itself deals with adversarial threat actors, exploitable vulnerabilities in complex and interconnected architectures, and the protection of confidentiality, integrity, and availability of data and system functions. Typical analytical methods include threat modelling, attack surface analysis, vulnerability assessment, and exploitability or impact assessments. A fundamental distinction from safety risk management is that the likelihood of a cybersecurity event is inherently difficult to quantify: adversaries act opportunistically and adaptively, threat landscapes shift with disclosure of new vulnerabilities, and historical frequency data rarely provides a reliable basis for probability estimation (6, 7). This leads to risk models that differ structurally from the probability-severity frameworks used in safety.

When safety and cybersecurity risks are managed within a single undifferentiated risk structure, heterogeneous risk types are forced into a shared information model and scoring logic. This approach creates several interrelated problems. First, it creates terminological confusion: safety risk estimation relies on probability of harm and severity, while cybersecurity assessment relies on exploitability, attack complexity, and impact on confidentiality, integrity, or availability. Forcing these into a common scoring matrix yields risk values that appear quantitatively comparable but carry no consistent analytical meaning. Second, although ISO 14971 expects the manufacturer to select risk analysis methods appropriate to the domain, Failure Mode and Effects Analysis has become the dominant technique in medical device risk management to the extent that alternative methods (fault tree analysis, event tree analysis, system-level threat modelling) are frequently underutilised or absent (8). This methodological narrowing leaves foreseeable event sequences, use-related hazards, and adversarial threat scenarios inadequately addressed. Third, undifferentiated structures offer no mechanism for conditional rigor: a minor cybersecurity observation may demand the same analytical workload as a life-threatening safety hazard, leading teams to either omit entries or produce superficial analyses that satisfy form without substance. These structural deficiencies are compounded when safety and cybersecurity risk activities are conducted by separate teams operating with independent risk registers, different review cycles, and no shared procedural reference within the quality management system. In the absence of a defined translation layer between the two domains, there is no systematic mechanism to escalate a cybersecurity finding to the safety process or to feed a safety constraint into threat modelling. The resulting consequences can include duplicated and siloed assessments, gaps in traceability between safety and security concerns, security measures added late in the development lifecycle as retrospective patches, safety and security controls that interact in unanticipated ways, and cross-functional teams burdened by frameworks that cannot capture what was actually decided, leading to disengagement from risk activities perceived as bureaucratic processes disconnected from real design decisions.

Regulatory bodies and standards organisations have clearly articulated the expectation that cybersecurity risk management must be coordinated with safety risk management and addressed within a total product lifecycle approach, while remaining analytically distinct. The IMDRF guidance states that cybersecurity risk management outcomes “should be aligned with the overall requirements of ISO 14971:2019, to ensure that output can be used as input for the overall risk management” (2). The FDA premarket guidance states that “performing security risk management is distinct from performing safety risk management as described in ISO 14971,” while simultaneously requiring that “security risk management should be an integrated part of a manufacturer’s entire quality management system, addressed throughout the TPLC” (6). MDCG 2019-16 identifies security as a component of the general safety and performance requirements under the EU Medical Devices Regulation (5). IEC 81001-5-1 provides a lifecycle process framework for health software security and explicitly states that security risk management “can be conducted under the framework of ISO 14971 with an appropriate mapping of vulnerability, threat and other security-related terms and addition of security-relevant activities,” (9). However, it does not itself define the structural or procedural mechanisms by which this mapping should be operationalised within a manufacturer’s quality management system. Collectively, these documents establish strong normative pressure toward integration but remain largely silent on the concrete methods, information structures, and procedural mechanisms by which manufacturers should achieve it. The gap is not one of intent but of operationalisation: manufacturers are told that safety and cybersecurity must connect, but not given a structural method for making that connection within their quality management systems and development toolchains.

This paper presents a modular risk schema framework designed to operationalise the integration of cybersecurity and patient safety risk management in the medical device software development lifecycle. The core principle is structural separation with explicit connection: safety and cybersecurity are treated as distinct risk domains, each with its own analytical model, vocabulary, and acceptability criteria, while a defined interface layer ensures that safety-relevant cybersecurity findings are systematically escalated and that safety constraints inform threat modelling. The framework accommodates multiple domain-specific risk schemas (including probability-severity for safety, exploitability-severity for cybersecurity, and additional schemas such as FMEA) operating in parallel within a single quality management system. It enforces conditional rigor through phase-gated deliverable controls aligned with the IEC 62304 software lifecycle, and integrates into modern Application Lifecycle Management environments while remaining tool-independent. The framework has been developed and applied in real-world medical device projects. The remainder of this paper is structured as follows: Section 2 details the architecture and operationalisation of the Modular Risk Schema Framework. Section 3 evaluates the framework against the problems identified above and sets out its limitations. Finally, Section 4 discusses its alignment with regulatory expectations and future extensibility.

## 2 Methods: modular risk schema framework

The risk management process underlying this framework follows the lifecycle defined in ISO 14971, risk analysis, risk evaluation, risk control, and evaluation of overall residual risk acceptability. The contribution of this work is not a new process but a structural and methodological layer that operationalises integrated safety and cybersecurity risk management within that established lifecycle: the Modular Risk Schema Framework. This section describes the framework’s architecture: how risk schemas are defined and differentiated, how they are connected through explicit traceability interfaces, and how the framework is embedded within a quality management system and realised in modern application lifecycle management environments.

### 2.1 Concept of risk schemas

Conventional approaches to medical device risk management typically employ a single, monolithic risk structure, a unified register or spreadsheet, into which all identified risks are recorded regardless of their nature or origin. In practice, this forces heterogeneous risk types into a shared information model: safety hazards, cybersecurity threats, component failure modes, and use-related risks all occupy the same fields, follow the same scoring logic, and pass through the same review workflow. Over time, such structures may exhibit schema drift as fields are renamed, repurposed, or left empty between projects, eroding the reliability of risk data across the product portfolio. The Modular Risk Schema Framework addresses this problem by introducing the concept of a *risk schema* as its fundamental building block. A risk schema is a structured specification for a family of risk items that share:

- a documentation rigor—the expected depth and formality of analysis;
- a risk model—the method by which risk level is judged or calculated;
- an information model—the fields and attributes that characterise each risk item;
- a review workflow—the states, transitions, and approval gates that govern the item’s lifecycle.

The documentation rigor component is the mechanism by which the framework supports conditional analytical effort: a schema applied at low rigor requires fewer mandatory fields and simpler review workflows than the same schema applied at high rigor, allowing the level of ceremony to be proportionate to the risk significance and regulatory demands of the product context.

In addition, each schema defines how its risk items link to items in other schemas and to lifecycle artefacts such as requirements, design elements, and verification records, as discussed in more detail in Section 2.4. Schemas are instantiated as individual risk items, and the totality of items across all active schemas forms the overall risk picture for a given product. A key principle enforced by the schema concept is atomicity: each failure mode, causal factor, effect, and control measure is captured as a distinct, individually traceable unit rather than embedded within overloaded free-text entries. This enables precise traceability, meaningful filtering and reporting, and reliable reuse of risk knowledge across projects.

### 2.2 Core schemas in the modular risk schema framework

As illustrated in Figure 1, the framework defines three core schemas that address the primary risk domains encountered in medical device software development: a Safety Risk Schema (SRS) for system-level safety risks, a Security Risk Schema (THREAT) for cybersecurity risks, and a Failure Mode and Effects Analysis schema (FMEA) for component-level failure analysis. These are not the only schemas the framework can support, but they represent the minimum configuration needed to achieve integrated safety and cybersecurity risk management.

**Figure 1:**
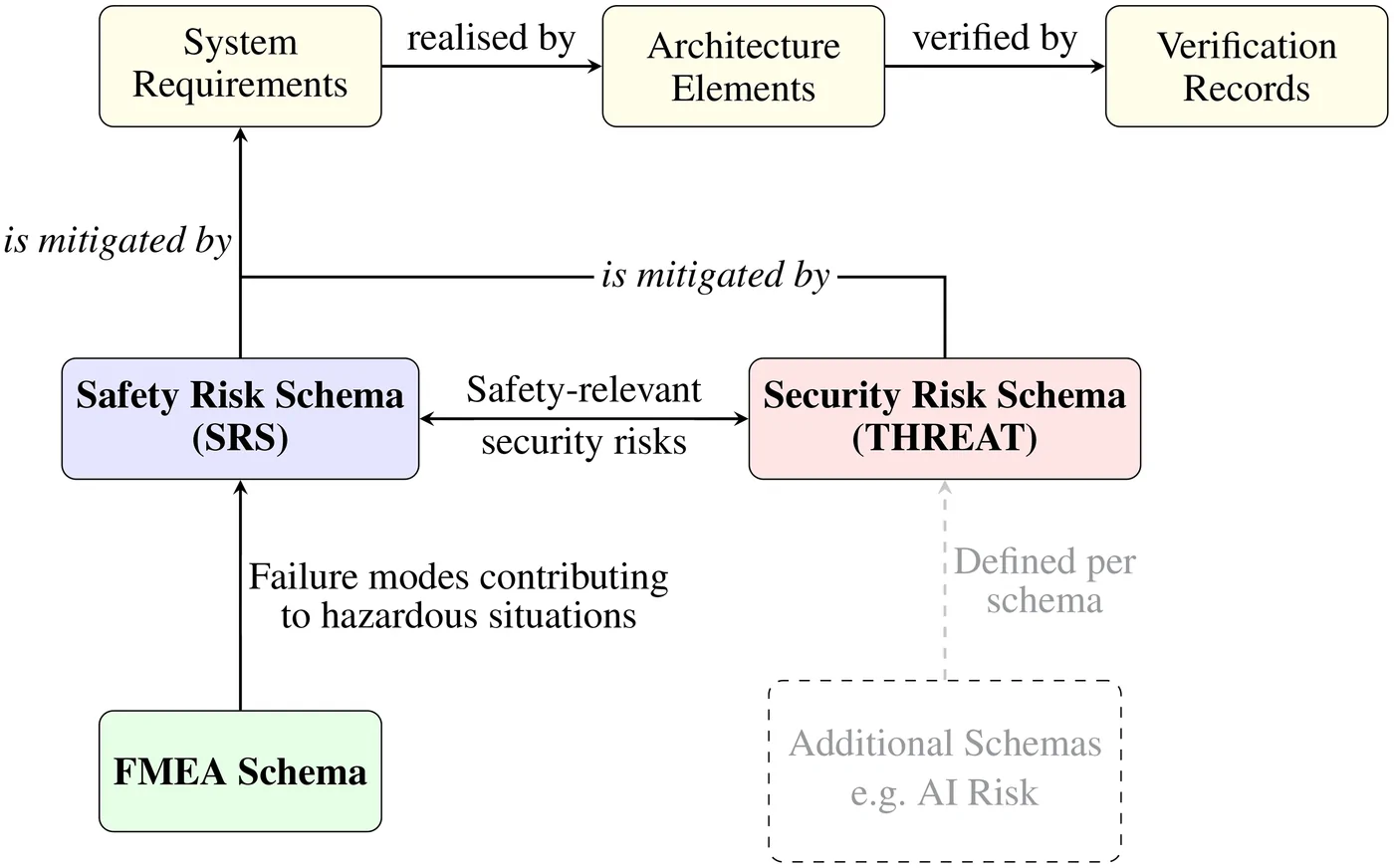
Schema architecture and traceability chain.

#### 2.2.1 Safety risk schema (SRS)

The Safety Risk Schema operates at the system level, combining top-down preliminary hazard analysis with structured identification of primary operating functions, environmental conditions, and clinical consequences. It adheres closely to ISO 14971 and serves as the principal instrument for understanding the overall risks and benefits associated with the device. Key concepts captured within SRS items include the device’s intended purpose, characteristics related to safety, identified hazards and hazardous situations, potential harms, external risk control measures, and the resulting safety classification. The SRS provides the authoritative view of whether residual safety risk is acceptable in relation to the expected clinical benefit.

#### 2.2.2 Security risk schema (THREAT)

The Security Risk Schema captures cybersecurity risks through a structured threat modelling approach. Each THREAT item is informed by identification of protected assets, identified threats, user roles, system architecture, third-party dependencies, end-points, module integration interfaces, and data flows. The schema supports evaluation of the implementation and completeness of the cybersecurity risk management plan, including threat and vulnerability identification, security verification activities, and post-market monitoring provisions. Where a security risk can contribute to or directly cause a hazardous situation, the THREAT item is linked to the corresponding SRS item—this is the primary mechanism by which cybersecurity findings are escalated to the safety process.

#### 2.2.3 Failure mode and effects analysis schema (FMEA)

The FMEA schema provides a complementary bottom-up perspective, focusing on specific failure modes of individual components or software units and their potential impact on device performance and safety. It is particularly suited to detailed technical scenarios where component-level behaviour must be understood, for example, analysing how a specific software module’s failure propagates through the system. The FMEA schema follows the conventional occurrence–severity–detectability model but benefits from the framework’s enforced atomicity and explicit linking to SRS and THREAT items where failure modes have safety or security implications.

#### 2.2.4 Extensibility with other schemas

The framework architecture is not limited to the three schemas described above. Additional schemas can be defined following the same structural pattern (information model, risk model, rigor, review workflow, and link definitions) whenever a domain requires its own analytical logic. This extensibility is a deliberate design choice: as regulatory landscapes evolve and new risk domains emerge, the modular architecture absorbs new requirements without destabilising schemas that already function effectively. Concrete candidates for future schemas are discussed in Section 4.4.

### 2.3 Distinct risk models and acceptance criteria per schema

A defining characteristic of the framework is that each schema operates with its own risk model and acceptance logic, tailored to the analytical demands of its domain. Forcing safety severity and cybersecurity exploitability into a single scoring matrix produces risk values that appear precise but carry no consistent analytical weight; the numbers become incommensurable. Similarly, risk acceptance criteria designed for one domain lose their meaning when applied to another with fundamentally different evaluation logic. The framework avoids this problem by allowing each schema to define domain-appropriate evaluation and acceptance mechanisms.

For the Safety Risk Schema, the risk model is based on the probability of hazardous situation(s) and severity of harm, expressed as a combination of P1 (probability that the hazardous situation occurs), P2 (probability that the hazardous situation leads to harm), and S (severity of the resulting harm). This aligns directly with ISO 14971 and is evaluated against manufacturer-specific risk acceptability criteria that reflect the intended clinical context and the available benefit-risk evidence.

The FMEA schema follows the conventional occurrence–severity–detectability model, in which risk priority is derived from the combination of how frequently a failure mode is expected to occur, how severe its consequences are, and how likely existing controls are to detect it before harm results.

Within the THREAT schema, classical probability estimation is replaced by attributes better suited to adversarial risk: exploitability (how readily the vulnerability can be weaponised given factors such as access requirements, complexity, and privileges needed) and timely observability (whether exploitation can be detected in time to respond), combined with severity of the potential impact. Where a security risk also has safety impact, it is linked to the Safety Risk Schema and evaluated from the safety perspective as well.

Exploitability and timely observability are scored on ordinal scales within the THREAT schema’s own model; the concrete scales, resulting risk levels, and acceptance criteria for that model are defined per product, as for the other schemas. Where a security risk is assessed to have no safety relevance, this internal model governs its evaluation and acceptance, following a product-defined rationale such as a documented cost-benefit judgement.

Where a security risk is escalated because it can contribute to a hazardous situation, its likelihood is instead computed within the safety risk model rather than within THREAT’s own model. Exploitability populates the probability that the hazardous situation occurs, and timely observability populates the probability that, once occurring, it leads to harm; the two are combined multiplicatively, mirroring the structure of the safety probability estimate. From this point, the escalated risk is evaluated under the existing safety acceptance criteria; no separate THREAT-level threshold applies.

Severity retains a dual role on escalation. The safety severity assessed within the linked SRS item governs the escalated risk, while the THREAT item continues to record its own security severity, such as loss of confidentiality, independently of that assessment.

Acceptance criteria for each schema can differ and are defined on a product- and project-specific basis. The framework accommodates this diversity by design: each risk domain should be assessed against domain-appropriate acceptance thresholds rather than forced into a single risk matrix. The framework enables these divergent risk models and criteria to coexist coherently within a single quality management system and to be fine-tuned according to project-specific needs without compromising overall consistency.

This separation has particular regulatory significance under the EU Medical Devices Regulation (MDR). MDR Annex I requires that safety risks be reduced as far as possible (AFAP) (10). In a monolithic risk structure, AFAP propagates implicitly to all risk items regardless of domain, producing either meaningless application (reducing exploitability “as far as possible” against an adaptive adversary) or empty compliance boilerplate. The modular architecture confines AFAP to the Safety Risk Schema where it carries regulatory force, while allowing the THREAT and FMEA schemas to operate under acceptance logic appropriate to their domains: product-defined acceptance rationale and engineering prioritisation, respectively.

### 2.4 Traceability interfaces between schemas and lifecycle artefacts

The modularity of the framework, which in practice means multiple schemas operating with distinct information models and risk logic, requires explicit mechanisms to ensure that the overall risk picture remains coherent and that findings in one domain are visible to others. The framework addresses this through defined traceability interfaces: structured links between risk items across schemas and between risk items and lifecycle artefacts. These interfaces are not optional documentation aids but mandatory structural elements that enforce escalation pathways and maintain analytical integrity across domains.

#### 2.4.1 Schema-to-schema links

The most critical traceability interface is the link between THREAT and SRS items. When a security risk identified in the THREAT schema can lead to or contribute to a hazardous situation, it must be linked to the corresponding SRS item. This is the mechanism by which cybersecurity findings are systematically escalated to system-level safety risk management, ensuring that safety analysts are aware of security-originated contributors to harm and can evaluate them within the benefit-risk framework of ISO 14971.

The interface operates bidirectionally. Safety controls defined in SRS or FMEA items that may affect cybersecurity (for example, fail-safe modes that alter system behaviour, alarm configurations that depend on network availability, or redundancy mechanisms that expand the attack surface) are linked back to relevant THREAT items. This ensures that security analysts can evaluate whether a safety-motivated design choice introduces new vulnerabilities or undermines existing security controls.

The framework also defines a traceability interface between the Safety Risk Schema and the FMEA schema. SRS operates at the system level and provides clinical consequences and hazardous situations as input to the FMEA, which then analyses how specific software item failure modes may contribute to those situations. The connection between the two schemas is ensured by tracing risk items based on the affected software item: each FMEA item that can contribute to a system-level hazardous situation links back to the corresponding SRS item. This vertical traceability—from system-level hazard down to component-level failure mode and back—ensures that bottom-up technical findings are systematically connected to their clinical significance.

Together, these schema-to-schema links form a network of explicit analytical relationships that prevent risks from existing in isolation. A finding in one schema cannot contradict or duplicate a conclusion in another without the relationship being visible and reviewable.

#### 2.4.2 Schema-to-lifecycle artefact links

Beyond schema-to-schema traceability, each risk item is linked to the lifecycle artefacts that implement or verify its associated controls. Risk control measures are linked to the system requirements that specify them, using defined issue relations: a risk item *is mitigated by* a system requirement (for controls internal to the device) or *is mitigated by* an external risk control (for controls external to the device). These requirements in turn trace forward to architectural elements that realise them and to verification records that confirm their effectiveness.

This supports the full traceability chain from identified risk through control measure to implementation and test, a chain that regulatory authorities expect to be demonstrable. The links are implemented within the Application Lifecycle Management (ALM) environment described in Section 2.6, using the tool’s native linking capabilities. The framework specifies what links must exist and what they mean; the specific implementation mechanism is left to the tooling choice.

### 2.5 Operationalising the framework within the quality management system and software lifecycle

The Modular Risk Schema Framework is not a standalone methodology but operates as an integral part of a Quality Management System. It is designed to be activated within the context of a product realisation project and requires product-specific inputs (clinical context, intended purpose, risk acceptability criteria) to function effectively. This positioning ensures that risk management activities are governed by the same document control, approval workflows, and audit mechanisms as all other quality processes.

#### 2.5.1 Risk management planning and documentation structure

For each product, the framework is instantiated through a defined documentation hierarchy. A product-specific risk management plan defines the scope of risk management, the schemas to be applied, assigned responsibilities and authorities, risk acceptability criteria, and the mapping of risk management activities to the software development lifecycle. The plan references the process-level definition but adds product-specific implementation decisions, such as which schemas are active, whether deviations from defaults are justified, and what clinical and technical inputs are required.

The risk management file serves as the container for all risk management records: the risk management plan, cybersecurity risk management plan, risk characterisation, risk management report, and cybersecurity risk management report. Together, these documents provide the evidence base for compliance claims: acceptable safety and security, acceptable benefit-risk ratio, and acceptable overall residual risk.

#### 2.5.2 Phase-gated secure-by-design enforcement

The framework enforces Secure-by-Design principles by aligning risk management activities with defined lifecycle phases. Rather than treating risk management as a monolithic front-loaded activity or a retrospective compliance exercise, specific deliverables and decisions are gated to specific phases of the software development process. Initial safety classification, preliminary risk characterisation, and initial threat analysis must be completed and reviewed before passing defined project milestones; later gates require updated risk items and integrated conclusions. The phase-gated approach is depicted in Figure 2.

**Figure 2:**
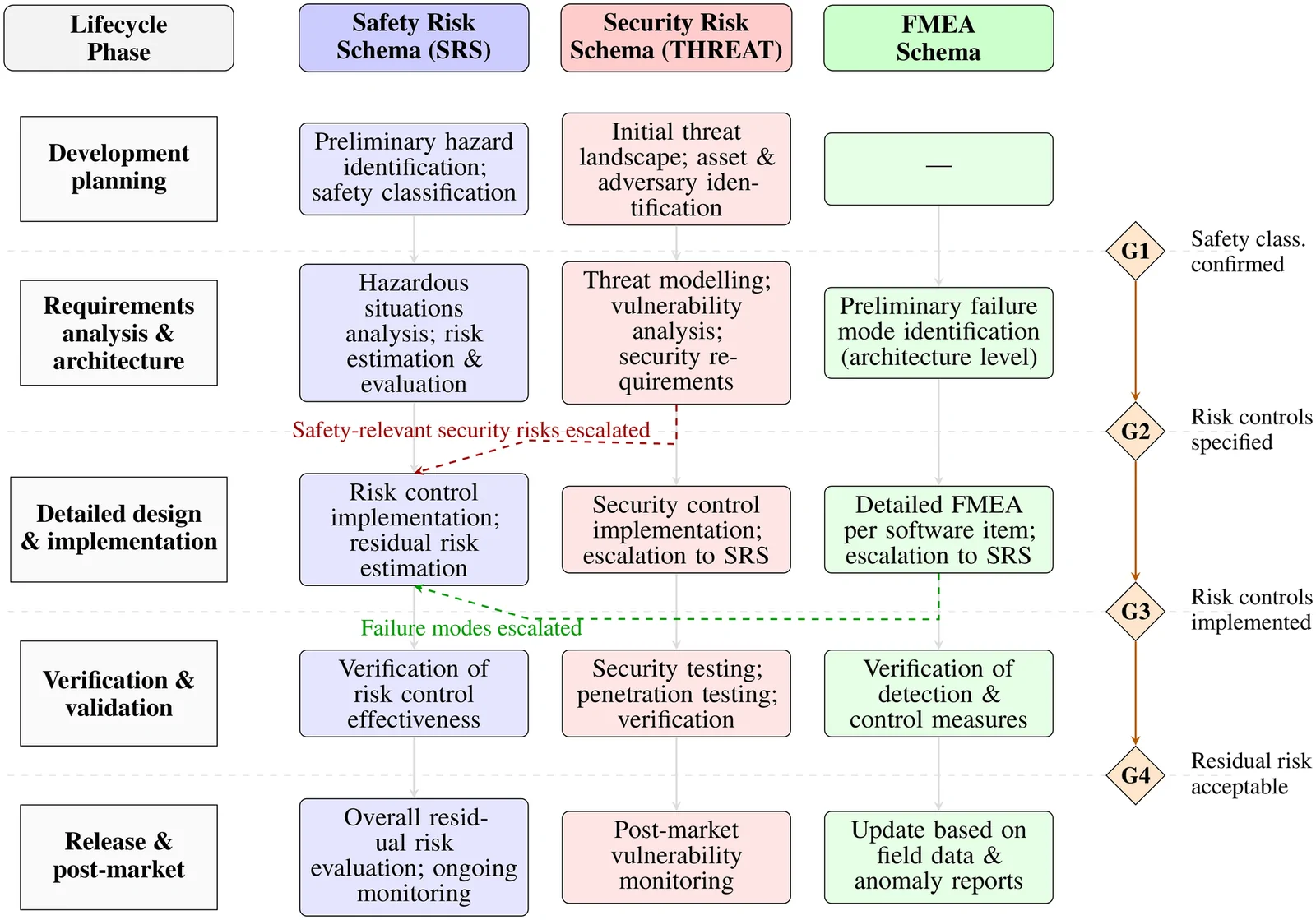
Phase-gated risk management activities aligned with the IEC 62304 software lifecycle.

This phase-gated structure serves two purposes. First, it ensures that security and safety considerations influence design decisions at the point where they can be addressed most effectively and at lowest cost. Second, it makes risk status visible at decision points, ensuring that development trade-off decisions are informed by the current safety and security landscape rather than made in isolation from it.

The framework does not, however, enforce a strict waterfall sequence. Activities may be conducted flexibly as the product evolves, provided that the logical dependencies between them are respected. For example, software safety classification may be provisionally assigned early in development and refined as the understanding of clinical consequences matures, but it must be finalised before it governs downstream development rigour decisions.

#### 2.5.3 Iterative risk management across the lifecycle

Risk management under this framework is understood as a continuous iterative process throughout the entire lifecycle of the device, not a one-time pre-market exercise. Post-market surveillance, newly identified threats and vulnerabilities, and changes to the device or its operating environment all trigger re-entry into the risk management process. The framework accommodates this by maintaining risk items as living artefacts within the ALM environment, subject to ongoing review, re-evaluation, and update as new information becomes available.

### 2.6 ALM implementation and tool independence

The framework specifies how schemas are concretely realised within Application Lifecycle Management environments while preserving independence from any specific tool. Most tools used for requirements traceability in medical device development also extend to risk management; the framework leverages this by requiring that risk items are managed within the same environment as requirements, architecture, and verification records.

#### 2.6.1 Mapping schemas to ALM structures

Each risk schema is mapped to a distinct item type in the ALM tool. The fields of a specific item type reflect the schema’s information model: mandatory and optional attributes correspond to the documentation effort defined for that schema, risk model numeric components are stored as numeric attributes to enable automatic risk level calculation, and the workflow states of each item type capture the review steps and approval gates specific to that schema.

This structural mapping ensures that the information model described in Section 2.1 is enforced by the tooling rather than relying on process description and analyst discipline. Without such enforcement, risk items in ALM environments tend to degenerate into overloaded free-text entries with no structural consistency, reproducing the problems of document-based approaches in digital form.

#### 2.6.2 Implementing traceability in the ALM environment

The traceability interfaces described in Section 2.4 are implemented using the ALM tool’s native linking capabilities. Risk items link to other risk items across schemas (THREAT to SRS, FMEA to SRS), and risk items link to the lifecycle artefacts that implement or verify their controls, such as system requirements, architectural elements, and test records. These links carry defined semantics: a risk item *is mitigated by* a system requirement or an external risk control; a THREAT item *is related to* an SRS item when the security risk contributes to a hazardous situation.

Risk management planning documents, risk characterisation records, and risk management reports are maintained within the same tool environment, functioning as a document repository co-located with the living risk items they summarise. This co-location ensures that the documentation remains synchronised with the evolving risk landscape rather than drifting out of alignment as the product develops.

#### 2.6.3 Tool independence

The framework emphasises tool independence: it specifies required capabilities rather than mandating specific ALM products. Any tool that can support multiple item types with configurable fields, numeric attributes for automatic calculations, configurable workflows with approval states, and typed links with defined semantics can implement the framework. This ensures that the methodology remains applicable across organisations with different tooling choices, and that migration between tools does not require re-engineering the risk management process, only re-mapping its structures.

### 2.7 Worked example: instantiating the framework

This section demonstrates the framework in operation through a single end-to-end example. It is a retrospective reconstruction based on a publicly documented cybersecurity finding, presented to show how the framework structures and escalates such a finding. It is not an account of how the manufacturer’s own risk management process operated, and it is not a critique of that process; the framework did not exist at the time of the events described. The risk model components are those defined by the schemas, but the scales and the values shown are illustrative instantiations rather than normative ones: the example demonstrates the structure and the escalation mechanics, while the scales, the number of risk levels, and the rule for deriving a level are defined per product.

#### 2.7.1 Case background

In 2015, independent security research established that a networked infusion pump could be reached over a hospital network and its drug library modified without authentication, because the device did not authenticate the communications it received (11). The drug library governs the upper and lower dose limits the pump enforces. An unauthorised modification could therefore alter the delivered dose of an infused therapy without any change visible to the clinician operating the device.

#### 2.7.2 Assessment within the THREAT schema

The finding is recorded as THREAT item THR-014. Table 1 shows the instantiated item. Exploitability is assessed as high: the configuration interface is reachable from the network segment the device is intended to operate on, requires no authentication, and needs no specialised tooling. Timely observability is assessed as low: no operational mechanism existed by which an unauthorised change to the drug library would produce a signal to clinical or technical staff, and the issue was identified by external research rather than by monitoring.

**Table 1:** Instantiated THREAT item, THR-014.

Attribute	Description
Protected asset	Integrity of the drug-library configuration governing dose limits
Identified threat	Unauthorised, unauthenticated network-based modification of infusion parameters
Attack surface	Remote configuration interface without authentication
Data flow	Hospital information system to pump: drug-library and dose-limit updates
Exploitability	High—unauthenticated access to the intended operating segment, standard tooling sufficient
Timely observability	Low—no operational mechanism signals an unauthorised configuration change
Security severity	Loss of integrity of a clinical configuration asset
Safety relevance	Yes—can contribute to a hazardous situation; escalation required

The item also records a security severity in its own right, reflecting loss of integrity of a clinical configuration asset. This assessment remains with the THREAT item throughout and is not the value that governs the escalated risk.

#### 2.7.3 Escalation to the safety risk schema

Because the threat can contribute to a hazardous situation, THR-014 is linked to SRS item SRS-007 (see Table 2) under the mandatory link type described in Section 2.4.1. Escalation is the point at which the substitution described in Section 2.3 applies. The probability components of SRS-007 are not estimated from frequency data, because no such basis exists for adversarial causes. Exploitability instead populates the probability that the hazardous situation occurs, and timely observability populates the probability that, once occurring, it leads to harm; the resulting probability is their product. Severity is assessed independently by the safety analyst on clinical grounds, and it is this safety severity, not the security severity retained in THR-014, that governs the escalated risk.

**Table 2:** Instantiated safety item, SRS-007, linked from THR-014.

Attribute	Description
Hazard	Loss of integrity of the infusion dosing configuration
Hazardous situation	Pump delivers a dose outside the clinically intended range following an undetected unauthorised configuration change
Harm	Death or serious injury from over- or under-infusion
Probability that the hazardous situation occurs	Substituted from the exploitability assessment in THR-014
Probability that harm follows	Substituted from the timely observability assessment in THR-014
Severity	Assessed on clinical grounds as severe: several common infusion therapies have narrow dosing margins, so a dose outside the intended range can be life-threatening
Evaluation	Not acceptable against the product’s safety acceptance criteria; risk control required before release

#### 2.7.4 Traceability to lifecycle artefacts

SRS-007’s risk control is mandatory authentication and integrity verification of drug-library configuration changes. This control is realised as system requirement SR-102: the device shall reject any configuration change that is not accompanied by a valid signature verifying its origin and integrity. The link between them is recorded as SRS-007 *is mitigated by* SR-102. SR-102 in turn traces forward to the architectural element that implements it, and to the verification record confirming that unsigned or unauthenticated update attempts are rejected.

System requirement SR-102 also illustrates the bidirectional interface between the two schemas. To satisfy it, the device reverts to its last validated configuration and raises a local alarm whenever verification fails. This fail-safe behaviour is itself a potential attack surface: repeated failed-verification attempts could force the device into a persistent lockout and interrupt therapy delivery. The framework records this as a new THREAT item linked back from SR-102, making a safety-motivated design decision visible to the security analyst instead of leaving it to be discovered in operation.

In summary, applying the framework in a product development context proceeds from planning, defining which schemas are active and at what rigor, through early risk characterisation, to ongoing management of living risk items in the ALM environment, culminating in integrated reports that state safety and security claims and evaluate overall residual risk. The example above traces one finding through that sequence. The following section evaluates the framework against the problems identified in the Introduction.

## 3 Results

This section evaluates the framework against the problems identified in the Introduction. Five criteria follow from those problems: whether domain vocabularies remain distinct, whether risk values retain their analytical meaning, whether analytical effort scales with risk significance, whether escalation between domains is structurally enforced, and whether risk documentation forms a coherent whole. A sixth criterion concerns the framework as a whole: which outcomes its structure obliges and which remain matters of analyst judgement. Each criterion is assessed against the framework’s structural properties and illustrated through the worked example of Section 2.7.

The evaluation is analytical rather than experimental. A controlled comparison, applying a monolithic and a modular approach to an identical product under identical conditions, is not feasible in regulated development: projects cannot be duplicated, teams cannot be blinded to methodology, and differences in product complexity, clinical context, and organisational maturity confound direct comparison. What can be established is what the framework’s structure requires and permits, which is what the criteria below address. Whether these structural properties improve development outcomes in measurable terms remains open, and metrics for that question are identified in Section 4.4.

### 3.1 Evaluation against identified problems

#### 3.1.1 Terminological confusion

Because each schema maintains its own information model (Section 2.1), safety and cybersecurity vocabulary is never forced into shared fields. Safety analysts work with hazardous situations, severity, and probability of harm; cybersecurity analysts work with threats, exploitability, and attack surfaces. In the worked example, THR-014 and SRS-007 describe the same underlying finding in the terms native to each domain, and neither item contains a field that would require one vocabulary to be expressed in the other. Severity illustrates the point most directly: the term appears in both schemas but denotes different things, the clinical consequence of harm in one and the consequence of a security compromise in the other, and the two are recorded as separate attributes rather than reconciled into a single value. Ambiguity is eliminated at the data-model level rather than managed through glossaries or naming conventions.

#### 3.1.2 Incommensurable risk values

Each schema applies a risk model appropriate to its domain: probability and severity for clinical hazards, exploitability and timely observability for adversarial threats, occurrence, severity, and detectability for component-level failure modes (Section 2.3). The resulting values are not brought into a common scale. A THREAT risk level orders findings within the security domain and is not compared against an SRS evaluation or an FMEA priority. In the worked example, THR-014 records its attributes and its own security severity while SRS-007 carries the safety evaluation, and no aggregate figure is computed across the two. Where a security risk does require evaluation in safety terms, the framework converts rather than compares: the escalated finding is assessed within the safety risk model itself, with exploitability and timely observability substituted into its probability components (Section 2.3). Each domain therefore retains its own analytical integrity, and cross-domain judgements are made by re-evaluating a finding under the receiving domain’s model rather than by placing two incompatible values side by side.

#### 3.1.3 Proportionality of analytical effort

The Introduction identifies a structural cause of superficial risk documentation: where a single register imposes uniform documentation demands, a minor security observation carries the same analytical workload as a life-threatening hazard, and teams respond either by omitting entries or by producing analyses that satisfy form without substance. The framework addresses this through documentation rigor as a schema property (Section 2.1). A schema applied at low rigor requires fewer mandatory fields and a simpler review workflow than the same schema applied at high rigor, and rigor is set per schema rather than per register, so effort scales with the analytical demands of the domain rather than with the most demanding item in the product. Within a schema, the risk level produced by that schema’s own model determines which items require detailed justification and higher review authority. What the structure cannot do is ensure that the effort spent is analytically productive; a team can meet a low-rigor field set as superficially as a high-rigor one. The mechanism removes a structural incentive toward superficial documentation without removing the possibility of it.

#### 3.1.4 No structural escalation mechanism

The link between a THREAT item and an SRS item is a mandatory element of the schema definition rather than a documentation convention (Section 2.4.1). Where a security finding can contribute to a hazardous situation, the link is required for the item to be complete, and its absence is detectable by automated validation of the data model rather than only by review. The worked example traces this: THR-014 is linked to SRS-007, and the escalation carries the exploitability and timely observability assessments into the safety evaluation rather than leaving the security finding to be described in safety terms by whoever notices it. The interface operates in both directions, so that a safety control with security implications reaches the security analyst by the same mechanism; in the example, the fail-safe behaviour required by SR-102 surfaces as a further THREAT item rather than as an unexamined consequence of a safety decision. What remains discretionary is the judgement that a finding is safety-relevant at all, which no link type can determine.

#### 3.1.5 Siloed assessments and the parallel document problem

Risk documentation is generated from the same underlying items rather than maintained as separate documents per discipline (Section 2.4). A safety report and a security report drawing on the same linked items cannot describe a finding differently without the discrepancy being visible in the items themselves, which is the condition that parallel documents maintained by separate teams do not satisfy. In the worked example, a single chain of items supports both the safety claim about SRS-007 and the security claim about THR-014, and the same requirement appears as the realisation of a safety control and as the origin of a security assessment. Phase gates additionally fix the points at which both analyses must be current (Section 2.5.2), so that a design decision at a gate is taken against the state of both domains rather than against whichever was most recently reviewed. Whether the items themselves are kept current is a matter of process discipline that co-location does not enforce.

#### 3.1.6 Structural enforcement vs. analyst discretion

The framework’s claims rest on what its structure obliges rather than on what a diligent analyst would do in any case. Three outcomes are enforced by construction. Domain vocabularies cannot merge, because they occupy separate information models rather than shared fields. A security finding with safety relevance cannot remain within the security domain, because the link to the safety schema is a mandatory element of the schema definition rather than a documentation convention. The AFAP obligation of MDR Annex I cannot propagate beyond the safety schema, because acceptance logic is a property of each schema rather than of the risk register as a whole. Section 2.7 shows each of these operating on a single finding. Other outcomes remain discretionary: whether a given security finding is judged safety-relevant in the first place, and whether the content of a linked item reflects genuine analytical reasoning, are matters of judgement that the structure records and exposes to review but does not determine. The boundary between the two is itself a finding: modularity constrains where analysis happens and what must connect to what, not the quality of the analysis performed.

### 3.2 Limitations and pitfalls

While the proposed framework addresses a recognised methodological gap, it is important to acknowledge its boundaries and potential implementation pitfalls.

#### 3.2.1 Validity constraints

The framework provides structure but does not replace domain expertise or expert judgement. Safety risk analysis requires clinical knowledge and understanding of hazardous situations; cybersecurity risk analysis requires threat modelling expertise and understanding of attack vectors. Risk acceptability decisions, benefit-risk evaluations, and the determination of whether a cybersecurity finding has safety relevance remain matters of human judgement that the framework makes visible and traceable but does not automate. ISO 13485 quality management system requirements mandate documented evidence of personnel competency for activities affecting product quality and safety, and the framework’s assignment of responsibilities in the risk management plan explicitly requires identified roles with appropriate qualifications. However, competency records confirm formal qualification but do not guarantee analytical depth. A person may satisfy documented competency requirements yet produce superficial analyses, particularly in cybersecurity where the threat landscape evolves rapidly and where practical experience with medical device threat modelling is still maturing as a discipline.

The framework enforces that the right fields exist and the right links are created, but it cannot enforce that the content within those fields represents genuine analytical reasoning. Teams under time pressure may satisfy structural requirements while providing minimal substantive analysis. The conditional rigor mechanism addresses this partially: risk items above certain thresholds require more detailed justification and higher review authority. However, this remains a human discipline problem that no structural framework can fully eliminate.

#### 3.2.2 Evidential basis

The framework has been developed in the course of medical device software development practice, but the projects concerned are subject to client confidentiality, and a documented account of their number, device classes, and observed outcomes cannot be published. The evaluation in Section 3.1 therefore rests on the framework’s structural properties and on the worked example rather than on that experience.

#### 3.2.3 Scope boundaries

The framework is designed and applied in the context of regulated medical device software development. Its full instantiation (SRS, THREAT, and FMEA with all associated traceability requirements and phase-gated deliverables) may introduce disproportionate overhead for products with lower regulatory burden. The modular architecture mitigates this by allowing partial instantiation. Because schemas are independent units with defined interfaces, organisations can select only those relevant to their context. A non-medical software product could adopt the THREAT schema and the FMEA schema without the safety schema, retaining the benefits of structured risk management (atomicity, conditional rigor, traceability) without the full regulatory ceremony.

#### 3.2.4 Implementation risks

Instantiating the framework (defining schemas, configuring item types, setting up workflows, and establishing link semantics) requires upfront investment and subject matter expertise in both risk management methodology and ALM tool configuration before any risk items are created. Once configured, however, schemas are reusable across products in a portfolio. Subsequent projects inherit the schema definitions and require only product-specific tailoring (scope, acceptability criteria, effort level).

If mandatory inter-schema links are not maintained, the escalation mechanism breaks down silently: findings in one domain that have relevance to another remain invisible without any explicit signal that information is missing. The framework supports automated data model validation; ALM environments can be configured to monitor compliance with mandatory link definitions and raise concerns when required traceability is absent, converting a silent failure into a visible one. However, while validation can enforce structural rules governing which item types may be linked and in what direction, it cannot assess whether a specific link target is analytically correct. An item linked to the wrong target, or linked superficially without genuine analytical connection, satisfies structural validation while still undermining the escalation mechanism.

## 4 Discussion

### 4.1 Positioning to existing approaches

#### 4.1.1 Cybersecurity frameworks and methods

Cybersecurity risk management is supported by a mature ecosystem of frameworks, yet these operate at different layers of abstraction and serve different purposes than the Modular Risk Schema Framework proposed in this work. Recognising this distinction clarifies that the framework is not a competitor to established cybersecurity methods but a structural layer within which they can operate.

A first group comprises governance and outcome frameworks such as NIST CSF 2.0 and ISO/IEC 27001. These organise cybersecurity activities into high-level functions or control objectives, providing a shared vocabulary for communicating risk posture across an organisation (12, 13). They define desired outcomes but do not prescribe the operational structures through which heterogeneous risk items are managed, connected, and traced at the engineering level. The modular schema framework operates below this governance layer: it provides the structural mechanism through which governance objectives are realised in day-to-day risk management practice.

A second group comprises risk assessment methodologies—ISO/IEC 27005, NIST SP 800-30 (14), OCTAVE (15), and the quantitative FAIR model (16)—which prescribe how security risk is identified, estimated, and evaluated. These methods are valuable for populating the analytical content of a security risk item, but they assume an information-security frame (confidentiality, integrity, availability) and typically a single risk register, and the quantification approach of methodologies such as FAIR conflicts with the difficulty of estimating adversarial likelihood noted earlier. The framework is agnostic as to which of these methods could inform the THREAT schema, treating the chosen technique as the analytical engine of a schema rather than as the organising structure of the overall risk picture. The same holds for threat-modelling techniques such as STRIDE (17), attack-tree analysis, and the adversary tactics catalogued in MITRE ATT&CK (18): these populate THREAT items but do not govern how those items relate to other domains.

A third group operates at the level of data representation and scoring. The Open Cybersecurity Schema Framework (OCSF) (19) provides a vendor-agnostic schema for normalising security events and telemetry, enabling interoperability across tools, while CVSS (20) standardises the severity scoring of individual vulnerabilities. These standards address the consistency of security data, not the management of risk across domains; OCSF can usefully inform the attribute structure of the THREAT information model, but it does not itself define acceptance criteria, conditional rigor, or escalation pathways.

#### 4.1.2 Safety risk management and integrated assessment methods

On the safety side, the positioning is different in nature. ISO 14971 provides both a process and an analytical vocabulary that are well established and not in need of replacement. The gap is not in safety risk management itself but in how it interfaces with adjacent domains. AAMI TIR57 (21) provides process-level guidance for coordinating security risk management with the ISO 14971 lifecycle, and IEC 81001-5-1 states that security risk management can be conducted under the framework of ISO 14971 with appropriate mapping, but neither defines the information structures, traceability mechanisms, or escalation pathways by which this coordination is concretely achieved.

Several published proposals integrate the two assessment processes directly. SAHARA (Security-Aware Hazard and Risk Analysis) combines safety hazard analysis with threat modelling within a single assessment process, deriving security levels using a determination matrix modelled on the automotive ASIL approach (22). While this achieves process-level integration, it assumes a domain-specific context and relies on structural parallels between safety integrity levels and security levels that do not transfer directly to medical device risk management under ISO 14971. FMVEA extends FMEA by treating the failure cause, failure mode, and failure effect model as a template for a corresponding vulnerability, threat mode, and threat effect chain, and by substituting an attack probability, derived from threat agent motivation and capability together with system reachability and unusualness, for the failure probability in the criticality assessment (23). STPA-SafeSec takes a control-theoretic route, extending STPA with a component layer that links the abstract control structure to the physical system design, and deriving safety and security constraints together so that the dependencies between cybersecurity vulnerabilities and system-level losses become explicit (24). A third proposal addresses a different aspect: independent safety and security analyses are performed first, combined into a co-analysis of each component through an explicit mapping between safety and security concepts, and used to derive fault trees from which interferences between the two domains are detected automatically, with the resulting reports serving to trigger co-engineering meetings and trade-off decisions (25).

#### 4.1.3 Technique and structure

These are analytical techniques: they produce findings. The framework presented here is a structure: it determines how findings, whatever technique produced them, are recorded, kept distinct, and traced through the regulatory documentation of a product. The techniques are therefore complementary to the framework rather than alternatives to it, and the distinction has two consequences.

The first concerns acceptance logic. The substitution of a security-appropriate probability into a safety-derived risk model is not itself the point of difference; the framework performs a comparable substitution when a security risk is escalated (Section 2.3). The difference lies in what the resulting value is evaluated against. Where the substitution feeds a single shared criticality measure, security and safety findings acquire values that appear comparable but rest on different foundations, a limitation acknowledged within FMVEA itself, where the attack probability estimate is noted as uncalibrated against failure probability and comparable results are said to require empirical attack data that is not available (23). The same integration also constrains severity: the FMVEA case study adopts a severity classification drawn from a dependability standard, which by its own account captures only consequences for dependability and excludes privacy as a factor (23). A related concern is raised from within the integrated approach in STPA-SafeSec, where a security finding without safety consequences, such as a breach of confidentiality causing loss to the operator, is argued to constitute a system loss in its own right rather than being relevant only through its safety impact (24). Under the present framework, the escalated finding is instead evaluated within the safety risk model against safety acceptance criteria, while the security severity remains recorded in the security schema, so that neither domain’s evaluation basis is required to absorb the other’s.

The second concerns what persists. The interference analysis approach is situated explicitly within the design stage, and its output is a report intended to trigger a co-engineering meeting rather than a maintained record (25). STPA-SafeSec is iterative and intended for reapplication across the lifetime of the system (24), but reapplication of an analysis is not the same as maintenance of the risk records that regulatory practice requires, in which each finding, control, and verification remains individually traceable across post-market changes and newly disclosed vulnerabilities. The framework addresses the persistence and traceability of risk information rather than the analysis that generates it.

The framework operates as a structural integration layer: it does not merge the assessment processes but preserves each domain’s native analytical method, providing the architecture within which the methods discussed above coexist and connect through explicit traceability interfaces.

### 4.2 Regulatory and standards alignment

The regulatory landscape for medical device cybersecurity has converged on a consistent position across jurisdictions: cybersecurity risk management must be analytically distinct from safety risk management yet integrated within the same quality management system and addressed throughout the total product lifecycle (2, 3, 5, 6, 9). However, none of these documents specify the structural or procedural mechanisms by which this “distinct yet integrated” position should be operationalised. The framework provides that operationalisation through its architecture of structural separation with explicit connection, as evaluated in Section 3.

ISO 14971 explicitly accommodates multiple risk analysis techniques and expects manufacturers to select those appropriate to the situation. In practice, however, this flexibility is easily eroded when organisations adopt a single template-driven approach, typically FMEA, and apply it indiscriminately regardless of analytical suitability. A risk management plan may name several techniques and still leave this unchanged, because a single undifferentiated register subjects every item to the same fields and the same scoring logic whatever the plan states. The framework makes methodological plurality structural rather than declarative: the risk management plan records which schemas are active for the product, and each active schema carries its own information model and analytical method in the tooling itself. The choice of technique is therefore visible in how risk items are constituted, not only in what the plan says about them. In particular, the separation prevents the AFAP obligation under MDR Annex I from propagating to risk domains where it has no defined meaning, while preserving its full force where it applies: within the Safety Risk Schema.

The FDA premarket cybersecurity guidance emphasises that effective cybersecurity relies upon security being “built in” to a device rather than “bolted on” after the device is designed (6). This Secure-by-Design expectation is reflected across IEC 81001-5-1 and MDCG 2019-16 as well, but none of these documents specify the procedural mechanism by which early integration is enforced. The framework’s phase-gated deliverable structure (Section 2.5.2) provides this mechanism, ensuring that security considerations influence design decisions at the point where they can be addressed effectively.

IEC 81001-5-1 and MDCG 2019-16 both acknowledge that security controls may conflict with safety requirements and vice versa. Without structural visibility into these interactions, trade-offs are resolved implicitly, or not resolved at all, creating latent contradictions in the risk management file. The framework’s bidirectional traceability (Section 2.4.1) makes these conflicts explicit and reviewable at defined points in the development lifecycle.

Finally, the framework provides a shared procedural reference within a single quality management system for disciplines that often operate in departmental silos with parallel risk activities and no mechanism to detect conflicts between them. This does not eliminate organisational specialisation but ensures that the outputs of safety and security analysis are structurally connected rather than merely co-located in time.

### 4.3 Organisational and cultural implications

The implications discussed in this section follow from the framework’s structure and describe expected consequences of adopting it; they are design rationale rather than observations from deployment.

The framework changes the interface between safety and cybersecurity disciplines. By requiring that safety-relevant cybersecurity findings are linked to system-level safety items, and that safety controls with security implications are visible to threat analysts, the framework creates structural occasions for cross-domain dialogue. A THREAT item linked to an SRS item is not merely a documentation artefact; it is a point where a security analyst and a safety analyst must agree on whether the connection is genuine and whether the safety consequence has been adequately addressed. These structured interaction points replace ad hoc coordination with repeatable, auditable exchanges.

This has implications for how responsibilities are assigned. The risk management plan must identify not only who performs each type of analysis but who is responsible for maintaining the links between schemas, that is, who ensures that escalation actually occurs. In practice, this often requires a role with sufficient understanding of both domains to recognise when a cybersecurity finding has safety relevance, or when a safety control introduces a new attack surface. The framework makes this responsibility explicit and assignable rather than leaving it as an implicit expectation that falls between organisational boundaries.

The framework also reframes the relationship between “Secure by Design” and “Safety by Design” from parallel slogans into a shared operational practice. When both disciplines operate within the same process structure, use the same ALM environment, and share defined traceability interfaces, the traditional separation between “security added the encryption” and “safety added the alarm” dissolves into a more integrated question: does this design decision serve the overall defensive posture of the device, and have its implications been evaluated from both perspectives? The phase-gated structure reinforces this by requiring that both safety and security analyses inform design decisions at the same milestones rather than in separate review cycles that may reach different conclusions about the same architectural choice.

However, structural visibility also surfaces disagreements that were previously hidden. A safety analyst and a security analyst may reach different conclusions about the same architectural decision—for example, whether a fail-safe mode that disables network connectivity adequately addresses a safety hazard while simultaneously creating an unmonitored security state. The framework does not resolve such disagreements; it makes them visible at defined points where they can be addressed through documented trade-off decisions rather than discovered late or never discovered at all. Organisations adopting the framework should expect an initial increase in visible cross-domain tensions, and should interpret this as evidence that the escalation mechanism is functioning rather than as a sign of dysfunction.

A practical consequence of this integration is that risk management activities become more meaningful to development teams. When risk items are structurally connected to the requirements, architecture, and verification records that developers work with daily, rather than existing in a parallel documentation universe maintained for compliance purposes, the perceived distance between “doing the work” and “documenting the risk” decreases. This does not guarantee engagement, but it removes one of the structural barriers to it: the sense that risk management is a separate bureaucratic activity disconnected from actual design decisions.

### 4.4 Future work

The modular architecture of the framework is designed to accommodate new risk domains through addition rather than restructuring. Several concrete extensions are anticipated as regulatory landscapes evolve and as the framework matures through further industrial application.

#### 4.4.1 Additional risk schemas for emerging regulatory domains

The EU AI Act and emerging harmonised standards introduce risk concepts, for example bias, transparency, robustness, and autonomous decision-making, that sit outside traditional safety and cybersecurity ontologies. For medical devices incorporating AI/ML components, these concerns require their own analytical vocabulary and acceptance logic: a bias risk cannot be meaningfully evaluated using a probability-severity matrix designed for physical harm, nor can transparency obligations be captured as cybersecurity threats. An AI-specific schema, following the same structural pattern as existing schemas, could capture these requirements within the framework without forcing them into safety or cybersecurity templates. Traceability interfaces to the Safety Risk Schema would ensure that AI-related risks with patient safety implications are escalated appropriately.

Similarly, data protection impact assessments follow a rights-and-freedoms logic that has no natural home in a safety-oriented risk process. The evaluation criteria (necessity, proportionality, risks to data subjects’ rights) differ fundamentally from both safety severity and cybersecurity exploitability. A DPIA schema with its own acceptance criteria and review workflow is a natural extension of the framework, particularly for devices that process sensitive health data. Links to the THREAT schema would capture the relationship between data protection risks and the cybersecurity controls that mitigate them.

#### 4.4.2 Generalisation beyond medical device software

The framework is currently designed for medical device software, but the structural principles it embodies are not inherently specific to this domain. Any context in which multiple heterogeneous risk types must be managed coherently within a single governance structure faces the same fundamental challenge that the framework addresses: how to preserve analytical integrity within each domain while ensuring that cross-domain implications are not lost.

Broader healthcare information systems and connected device ecosystems are immediate candidates. A hospital information system that processes clinical data, controls medical device integrations, and manages patient-facing services simultaneously faces safety, cybersecurity, data protection, and operational continuity risks—each with distinct analytical logic and acceptance criteria. The framework’s architecture could accommodate these as parallel schemas connected through defined traceability interfaces, providing structural coherence without forcing incommensurable risk types into a shared model.

Beyond healthcare, safety-critical software domains such as automotive, industrial automation, and aviation face analogous regulatory pressure to integrate functional safety with cybersecurity. The specific schemas would differ, but the meta-level architecture transfers directly. Evaluating this applicability through concrete instantiation in other domains is a natural direction for further work.

#### 4.4.3 Reusable risk knowledge across products

Currently, each product development project typically begins its risk analysis from a blank state, even when the organisation has previously analysed similar architectures, clinical contexts, or technology stacks. This repetition is partly a consequence of how risk information is traditionally stored: embedded in document prose or unstructured spreadsheet entries that cannot be meaningfully queried or reused.

The framework’s enforced atomicity and structured traceability create a precondition for changing this. Fully traced risk chains, from software items through causal factors to hazardous situations, with associated controls and verification evidence, are stored as discrete, linked items rather than narrative text. A mature ALM implementation could expose these chains as reusable organisational assets: when a new product employs a similar software component or clinical function, previously analysed risk chains could serve as starting points rather than requiring de novo analysis. This would not eliminate the need for product-specific evaluation, but it would reduce the effort required to reach a baseline risk characterisation and would help preserve institutional knowledge that is otherwise lost when personnel change between projects.

#### 4.4.4 Empirical validation and tooling maturity

The framework has been developed and applied in industrial medical device projects, but its effectiveness has not yet been evaluated quantitatively. Metrics of interest include traceability completeness, analytical effort relative to project complexity, time from cybersecurity finding to safety escalation, and defect escape rates for issues that cross domain boundaries. A controlled comparison between projects using the framework and projects using conventional approaches would provide empirical evidence for the claimed benefits, though the practical challenges of such a comparison in regulated development contexts are considerable.

The practical effort of instantiating the framework could also be reduced through further tooling maturity: schema templates that pre-configure ALM item types for common product categories, automated link validation that continuously monitors traceability completeness, and compliance dashboards that provide real-time visibility into the state of risk management across a product portfolio. These capabilities are enabled by the framework’s structured approach but require investment to realise fully.

## 5 Conclusions

The increasing connectivity of medical device software demands risk management approaches capable of addressing both traditional safety hazards and evolving cybersecurity threats, without compromising the integrity of either discipline. Unfortunately, attempting to merge safety and cybersecurity into a single unified risk model introduces significant practical and conceptual limitations, including inconsistent terminology, incomparable risk estimations, and weak traceability between domains.

In this paper, we have proposed the Modular Risk Schema Framework that addresses the above challenges by enabling independent yet interconnected safety and cybersecurity risk management approaches within a shared quality management system. By preserving domain-specific vocabularies, risk models, acceptance criteria, and assessment methodologies, the framework avoids artificial normalisation of fundamentally different risk concepts while still ensuring coordinated decision-making through explicit traceability interfaces and escalation mechanisms. Moreover, the proposed framework operationalises Secure by Design principles through lifecycle-aligned phase gates and structured deliverables that integrate with contemporary software development and application lifecycle management practices. Its implementation-oriented structure supports scalability, repeatability, and regulatory alignment without dependence on proprietary tooling or a specific organisational structure.

The framework’s structure is compatible with standards such as ISO 14971, IEC 62304, and IEC 81001-5-1, as well as current medical device cybersecurity guidance. Hence, the presented approach provides a practical foundation for organisations seeking to systematically integrate safety and cybersecurity risk management in modern medical device software development environments.

## Data Availability

The original contributions presented in the study are included in the article/Supplementary Material, further inquiries can be directed to the corresponding author.
